# Knockout of cytochrome P450 1A1 enhances lipopolysaccharide‐induced acute lung injury in mice by targeting NF‐κB activation

**DOI:** 10.1002/2211-5463.12977

**Published:** 2020-09-23

**Authors:** Li‐xing Tian, Xin Tang, Wei Ma, Jing Wang, Wei Zhang, Kuan Liu, Tao Chen, Jun‐yu Zhu, Hua‐ping Liang

**Affiliations:** ^1^ State Key Laboratory of Trauma, Burns and Combined Injury Department of Wound Infection and Drug Daping Hospital Army Medical University Chongqing China; ^2^ Department of Intensive Care Unit the Affiliated Hospital of Zunyi Medical University Zunyi China; ^3^ Department of Emergency The Third Affiliated Hospital of Chongqing Medical University Chongqing China; ^4^ Emergency and Trauma College Hainan Medical University Haikou China

**Keywords:** acute lung injury, cytochrome P450 1A1, LPS, NF‐κB

## Abstract

Acute lung injury (ALI) is accompanied by overactivation of multiple pro‐inflammatory factors. Cytochrome P450 1A1 (CYP1A1) has been shown to aggravate lung injury in response to hyperoxia. However, the relationship between CYP1A1 and lipopolysaccharide (LPS)‐induced ALI is unknown. In this study, CYP1A1 was shown to be upregulated in mouse lung in response to LPS. Using CYP1A1‐deficient (CYP1A1−/−) mice, we found that CYP1A1 knockout enhanced LPS‐induced ALI, as evidenced by increased TNF‐α, IL‐1β, IL‐6, and nitric oxide in lung; these effects were mediated by overactivation of NF‐κB and iNOS. Furthermore, we found that aspartate aminotransferase, lactate dehydrogenase, creatine kinase, and creatinine levels were elevated in serum of LPS‐induced CYP1A1−/− mice. Altogether, these data provide novel insights into the involvement of CYP1A1 in LPS‐induced lung injury.

## Graphical abstract

CYP1A1 was induced in lung of mice by LPS treatment with an aryl hydrocarbon receptor‐independent manner, while Toll‐like receptor 4 knockout abolished CYP1A1 overexpression. CYP1A1 deficiency elevated pro‐inflammatory cytokines in lung tissues of septic mice by targeting NF‐κB (p65) and increased lung injury in response to LPS or *E*.* coli* challenge.

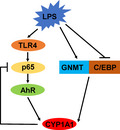

**Abbreviations**
AhR: aryl hydrocarbon receptor
ALI: acute lung injury
ARDS: acute respiratory distress syndrome
CYP1A1: cytochrome P450 1A1
EETs: epoxyeicosatrienoic acids
LPS: lipopolysaccharide
TLR4: Toll‐like receptor 4

Acute lung injury (ALI) and acute respiratory distress syndrome (ARDS) are serious diseases of the lung characterized by overexpression of multiple pro‐inflammatory cytokines. ALI and ARDS are responsible for respiratory failure in ICU patients [1]. ALI has several direct and indirect causes but bacterial infectious diseases, for example, sepsis, are the major indirect cause, resulting in impaired lung function, immoderate pro‐inflammatory factor expression, neutrophil infiltration, and alveolar destruction [2]. Lipopolysaccharide (LPS), a component of *Escherichia coli*, plays an important role in sepsis‐induced ALI by triggering a pro‐inflammatory cascade via NF‐κB activation and subsequent cytokine production [3]. Although a number of pharmacological interventions exist for ALI, mortality remains unacceptably high [4]. Thus, it is essential to identify novel therapeutic targets and effective pharmacological treatments for patients with ALI.

Cytochrome P450 1A1 (CYP1A1) is a monooxygenase regulated by the inflammation‐limiting aryl hydrocarbon receptor (AhR) [5], mainly involved in the metabolism of a broad spectrum of xenobiotics [6, 7, 8]. Emerging evidence demonstrates CYP1A1 to participate in lung inflammatory responses. Upregulated CYP1A1 in pulmonary alveolar macrophages has been shown to suppress major inflammatory cytokine production induced by *Mycoplasma* through elevation of PPAR‐γ [9]. Another study demonstrated mice deficient in CYP1A1 to be more susceptible to hyperoxic lung injury, although the mechanistic basis was unclear [10]. CYP1A1 has been shown to be upregulated in the lungs of incense‐induced mice, accompanied by an increase in multiple pro‐inflammatory factors [11]. Although these results collectively indicate a potential role for CYP1A1 in regulation of lung injury, no studies have investigated the relationship between LPS‐induced ALI and CYP1A1.

In this study, we found CYP1A1 to be highly expressed in mouse lungs following LPS stimulation. CYP1A1 was identified as an important regulator of pro‐inflammatory factors in ALI by increased activation of NF‐κB and iNOS. Furthermore, we demonstrated for the first time that a CYP1A1 deficiency impedes mouse hepatic, renal, and cardiac function in response to LPS. Taken together, these results describe a novel CYP1A1‐related signaling pathway, CYP1A1‐NF‐κB‐iNOS, which may be a promising target for ALI treatment, especially during sepsis.

## Materials and methods

### Reagents

Lipopolysaccharide (*E*. *coli* 0111: B4) was purchased from Sigma‐Aldrich (St. Louis, MO, USA). Pyrrolidinedithiocarbamate ammonium (PDTC) was obtained from Selleck Chemicals (Houston, TX, USA). PBS was provided by Boster (Wuhan, Hubei, China).

### **Preparation of *E***.*** coli* cells**

*Escherichia coli* cells were obtained from ATCC (Manassas, VA, USA) and cultured at 37 °C for commonly maintaining in our laboratory.

### Mice and treatment

Wild‐type (WT) C57BL/6 mice (male, 10–12 weeks, weighing 20–25 g) were obtained from the Experimental Animal Center of the Army Medical University (Chongqing, China). AhR knockout (AhR−/−) and normal (AhR+/+) mice were purchased from Jackson Laboratory (Bar Harbor, ME, USA). CYP1A1 knockout (CYP1A1−/−) and normal (CYP1A1+/+) mice were provided by GemPharmatech (Nanjing, Jiangsu, China). Toll‐like receptor 4 knockout (TLR4−/−) and normal (TLR4+/+) mice were kindly provided by X. Xu (State Key Laboratory of Trauma, Burns, and Combined Injury, Daping Hospital, Army Medical University, Chongqing, China). These mice were raised in isolation with specific pathogen‐free conditions. All experimental procedures and animal welfare protocols were conducted in accordance with the guidelines for laboratory animal care of the National Institutes of Health and Army Medical University. Mice were intraperitoneally injected with LPS at a dosage of 15 mg·kg^−1^ to induce ALI, based on a previous study [3]. Mice were intratracheally instilled with *E*.* coli* (CFU 5 × 10^8^/40 μL) to induce bacterial ALI [12]. Mice were anesthetized before sacrificing by using phenobarbital.

### Western blot

The lung tissues were harvested from treated mice at the indicated times, homogenized with a glass homogenizer, maintained on ice for 30 min, and then centrifuged at 12 000 ***g*** for 15 min at 4 °C. Extracted proteins were quantified using a bicinchoninic acid assay kit (Beyotime, Wuhan, Hubei, China) following a standard protocol. Equal amounts of protein (60 μg) per gel lane were separated by 10% SDS/PAGE and transferred to polyvinylidene difluoride membranes. Transferred membranes were blocked with 5% skim milk for 1.5 h at room temperature (RT) and incubated with target primary antibodies overnight at 4 °C. The blotted membranes were flushed with Tris‐buffered saline with Tween 20 and incubated with secondary antibodies for 1 h. The immunoreactive bands were visualized using an enhanced chemiluminescence detection system (Bio‐Rad, Hercules, CA, USA). Primary antibodies reactive with AhR (rabbit mAb; cat. no. 83200), GAPDH (rabbit mAb; cat. no. 5174), iNOS (rabbit mAb; cat. no. 13120), phosphorylated p65 (rabbit mAb; cat. no. 3033), total p65 (rabbit mAb; cat. no. 8242), phosphorylated IκB‐α (rabbit mAb; cat. no. 2859), total IκB‐α (rabbit mAb; cat. no. 4812), phosphorylated STAT1 (rabbit mAb; cat. no. 9167), total STAT1 (rabbit mAb; cat. no. 14994), phosphorylated jun (rabbit mAb; cat. no. 2361), total jun (rabbit mAb; cat. no. 9165), phosphorylated fos (rabbit mAb; cat. no. 5348), and total fos (rabbit mAb; cat. no. 2250) were purchased from CST (Beverly, MA, USA) and used at the dilution 1 : 1000. CYP1A1 (rabbit PcAb; cat. no. 13241‐1‐AP) was obtained from Proteintech (Chicago, IL, USA) and used at the dilution 1 : 1000. Secondary antibodies (goat anti‐rabbit IgG; cat. no. 7074) were produced by CST and used at the dilution 1 : 1000 for both western blot and immunohistochemistry assessments.

### Lung/body weight ratio

Before sacrifice, treated mice were weighed, and after sacrifice, their lungs were weighed. The ratio of lung to body weight was calculated.

### Wet/dry weight ratio

To assess the degree of lung edema, wet/dry weight ratios were calculated. Lungs were weighed and maintained in an oven at 80 °C for 24 h and then weighted again. The ratio of wet weight to dry lung weight was calculated as wet/dry ratio.

### Histologic examination

The lungs were flushed with PBS to remove excessive blood and then fixed with 4% paraformaldehyde for 15 h at RT. The tissues were blocked in paraffin and sectioned at 5 μm thickness. The sectioned tissues were stained with hematoxylin and eosin (H&E) staining after deparaffinization and rehydration to assess morphological changes.

### Cytokine assessment

The concentration of TNF‐α, IL‐1β, and IL‐6 in lung homogenates and bronchoalveolar lavage fluids (BALF) was measured by ELISA (Boster, Wuhan, Hubei, China) according to standard protocols. The levels of nitric oxide (NO) in lung homogenates and BALF were determined with a (NO) detection kit (Beyotime) following the manufacturer's instructions.

### Myeloperoxidase (MPO) activity

Lung tissues were harvested after treatment, flushed with cold PBS, homogenized, and centrifuged. Supernatants were assessed for MPO activity using a MPO activity detection kit (Boster) according to standard protocol.

### Immunohistochemistry

The lungs of LPS‐treated mice were fixed with 4% paraformaldehyde for 15 h at RT. The fixed lungs were embedded in paraffin and sectioned at 5 μm thickness. The deparaffinized sections were treated with 0.3% hydrogen peroxide in 60% methanol for 30 min to block endogenous peroxidase activity and then permeabilized with 0.1% Triton X‐100 in PBS for 20 min. The sections were incubated in 3% goat serum in PBS for 20 min and then blocked with avidin and biotin. The processed sections were maintained in iNOS reactive antibody (CST; rabbit mAb; cat. no. 13120; 1 : 200) or control solution overnight at 4 °C. A biotin‐conjugated specific secondary anti‐immunoglobulin G and avidin–biotin peroxidase complex were used to detect specific binding. To confirm the specificity of the iNOS antibody, some sections were incubated with primary antibody (in the absence of secondary antibody) or with secondary antibody (in absence of primary antibody). No positive staining was observed for these sections.

### NF‐κB p65 DNA‐binding activity

Treated lungs were lysed with a nuclear and cytoplasmic protein extraction kit (Beyotime). The NF‐κB p65 DNA‐binding activity in lung homogenates was assessed using a TransAM NF‐κB p65 transcription factor assay kit (Active Motif, Carlsbad, CA, USA) according to the standard instructions.

### Aspartate aminotransferase (AST), alanine aminotransferase (ALT), lactate dehydrogenase (LDH), creatine kinase (CK‐MB), and creatinine (Crea) measurement

Blood samples were separated by centrifugation (300 ***g***, 10 min) and the serum portion sent to the Department of Laboratory Medicine, Daping Hospital, Army Medical University, for quantification of AST, ALT, LDH, CK‐MB, and Crea.

### Statistical analysis

Most data are presented as the mean ± SEM. Continuous variables were compared between two groups by two‐tailed Student′s *t*‐test. All statistical analyses were performed using spss 16.0 (SPSS Inc., Armonk, NY, USA) or prism 6.0 (GraphPad software Inc., La Jolla, CA, USA) software. A *P*‐value < 0.05 was considered statistically significant.

## Results

### CYP1A1 is elevated in the lungs of LPS‐induced mice in an AhR‐independent manner

**Fig. 1:**
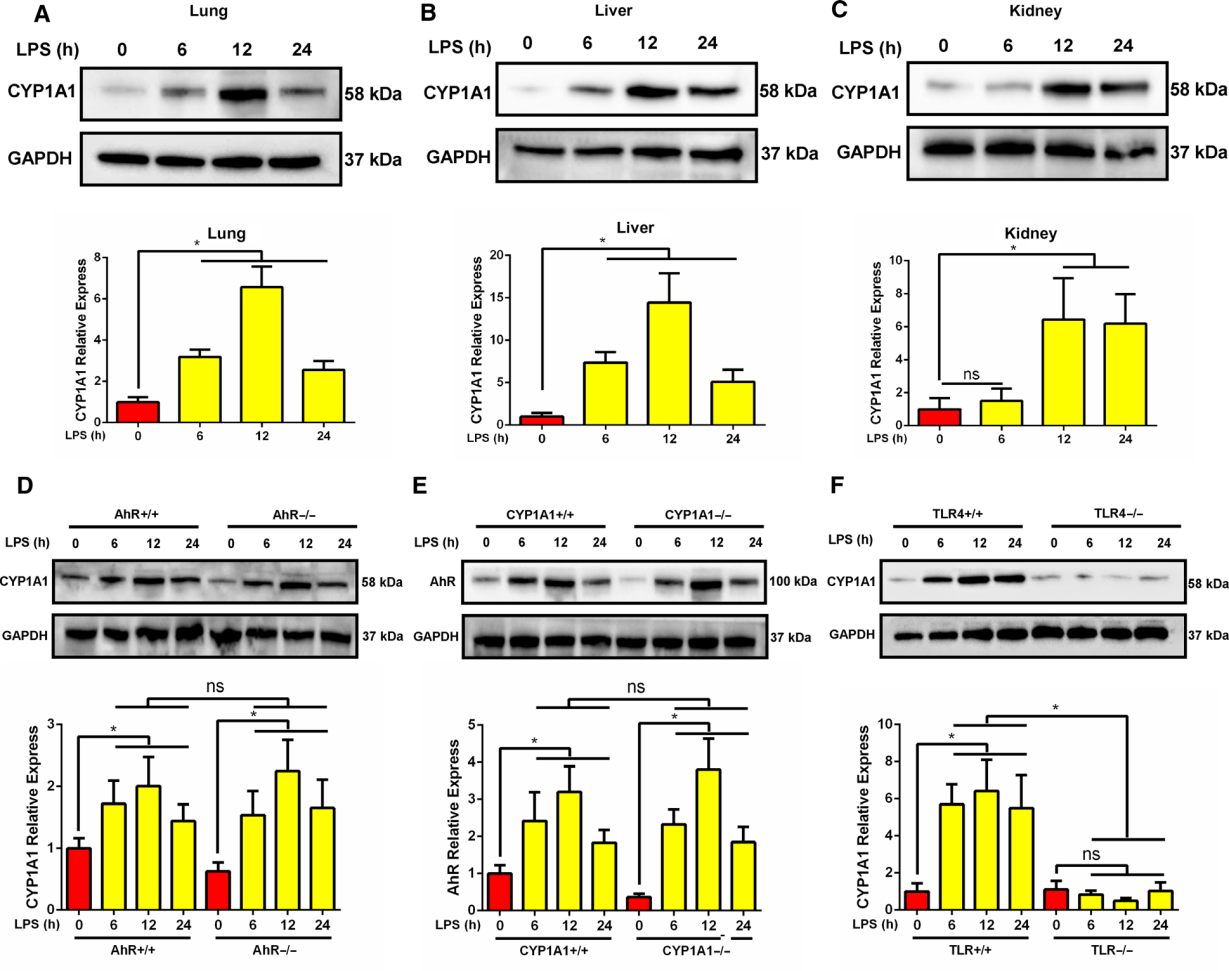
CYP1A1 is elevated in lungs of LPS‐induced mice in an AhR‐independent manner. (A‐C) WT mice were intraperitoneally injected with LPS (15 mg·kg^−1^) for 6, 12, and 24 h. The lungs, liver, and kidneys of treated mice were harvested at the indicated times for CYP1A1 protein measurement by western blot analysis and quantified by densitometry. (D–F) Normal, AhR−/−, CYP1A1−/−, and TLR4−/− mice were intraperitoneally injected with LPS for 6, 12, and 24 h. The lungs of treated mice were obtained at the indicated times for CYP1A1 and AhR protein measurements by western blot analysis and quantified by densitometry. Group means were compared by Student's *t*‐test. Data shown are means ± SEM. *P < 0.05. ns, no statistical difference.

To assess expression levels of CYP1A1, WT mice were intraperitoneally injected with LPS. As shown in Fig. 1A–C, we found CYP1A1 to be upregulated at 6 h with a peak at 12 h after LPS treatment in lungs, livers, and kidneys. As AhR is the main regulator of CYP1A1, we observed that AhR protein levels were remarkably elevated in lungs from LPS‐treated mice (Fig. 3E). However, the levels of CYP1A1 were still upregulated in lungs from LPS‐induced AhR−/− mice (Fig. 1D). We also found that AhR protein levels were still highly overexpressed in lungs from LPS‐treated CYP1A1−/− mice (Fig. 1E). These data indicate a possible AhR‐independent regulation of CYP1A1 in the progression of LPS‐induced ALI. LPS is a specific ligand for mediating TLR4‐related cascade reactions, including NF‐κB activation, which is involved in the expression of AhR in macrophages [13, 14, 15]. Herein, we investigated the relationship between CYP1A1 and TLR4. As shown in Fig. 1F, TLR4 knockout significantly reduced CYP1A1 expression in response to LPS challenge. These results indicate that CYP1A1 expression in the lungs of LPS‐induced mice is TLR4‐dependent and not AhR‐dependent, since CYP1A1 knockout has no effect on AhR expression. These data also provided a possible strategy for regulation of CYP1A1 during ALI.

### CYP1A1 deficiency aggravates LPS‐induced lung injury in mice

**Fig. 2:**
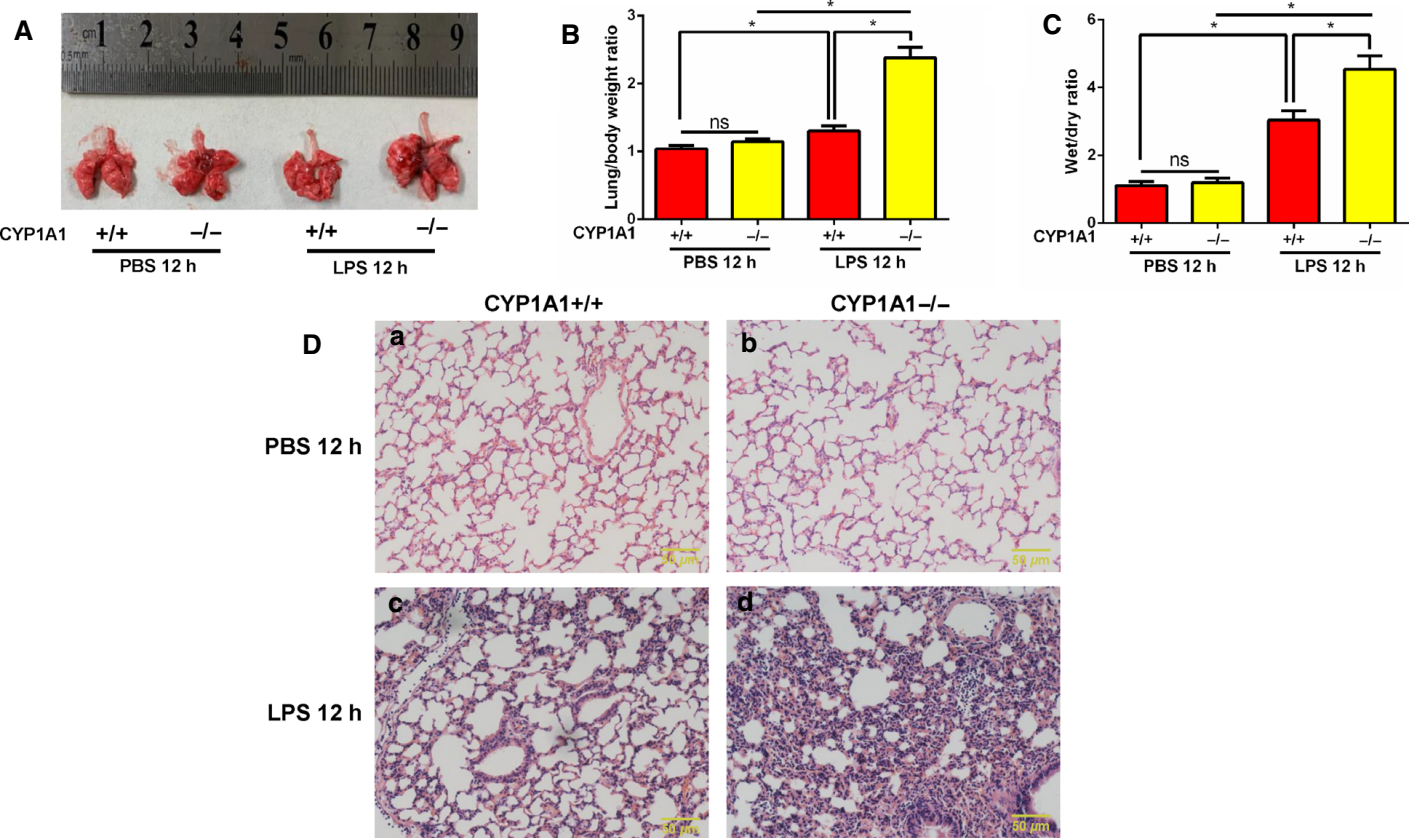
CYP1A1 deficiency aggravates LPS‐induced lung injury in mice. (A–D) CYP1A1+/+ and CYP1A1−/− mice were intraperitoneally injected with PBS or LPS (15 mg·kg^−1^) for 12 h after which lungs were removed. (A) Lung photographs. (B) Lung weight/body weight ratios were quantified (*n* = 5 for each group). (C) Wet‐to‐dry ratios of lungs were quantified (*n* = 5 for each group). (D) Sections of lungs were stained with H&E. Bar: 50 μm. Group means were compared by Student's *t*‐test. Data shown are means ± SEM. *P < 0.05. ns, no statistical difference.

CYP1A1+/+ and CYP1A1−/− mice were intraperitoneally injected with LPS for 12 h, sacrificed, and lungs harvested. As shown in Fig. 2A, by gross appearance the size of lungs from LPS‐induced CYP1A1−/− mice was larger than that of LPS‐induced CYP1A1+/+ mice. The lung/body weight ratio was increased in CYP1A1−/− compared to CYP1A1+/+ mice (Fig. 2B). The wet/dry ratio (W/D) of lungs from the CYP1A1‐deficient mice was higher at 12 h after LPS challenge when compared to CYP1A1+/+ mice treated with LPS (Fig. 2C), indicating that CYP1A1−/− lung tissue exhibited edema. Histology (H&E staining) was used to directly observe changes in the lungs of LPS‐induced CYP1A1−/− mice. As shown in Fig. 2D (a,c), LPS treatment induced conspicuous injuries in lung tissues, including extensive alveolar wall thickening, neutrophil infiltration, and alveolar collapse, while these morphologic alterations were remarkably strengthened by CYP1A1 deficiency in response to LPS when compared to LPS‐induced CYP1A1+/+ mice (Fig. 2D: c,d). These results directly demonstrate the regulatory effect of CYP1A1 in LPS‐induced ALI.

### CYP1A1 deficiency enhances LPS‐induced pro‐inflammatory factor levels in the lung

**Fig. 3:**
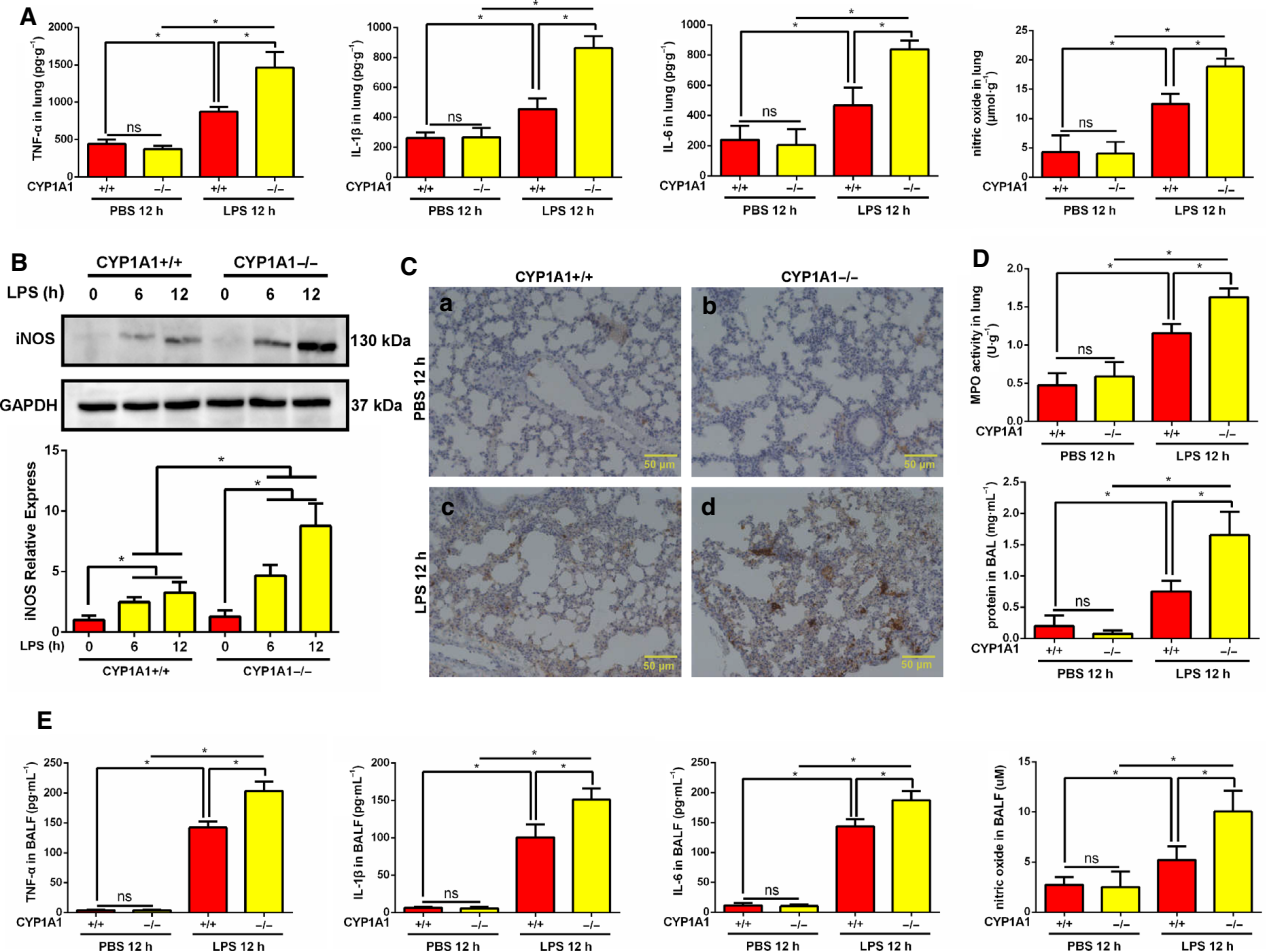
CYP1A1 deficiency enhances LPS‐induced pro‐inflammatory factor levels in lung. (A, C‐E) CYP1A1+/+ and CYP1A1−/− mice were intraperitoneally injected with PBS or LPS (15 mg·kg^−1^) for 12 h. (A) TNF‐α, IL‐1β, IL‐6, and NO levels were measured in lung homogenates of treated mice by ELISA (*n* = 5 for each group). (B) The lungs were harvested after 6 and 12 h LPS treatment for iNOS protein assessment by western blot and quantified by densitometry. (C) Lung tissue immunohistochemical staining. Bar: 50 μm. (D) MPO activity in lung homogenates and total protein levels in BALF, respectively (*n* = 5 for each group). (E) TNF‐α, IL‐1β, IL‐6, and NO levels assessed in BALF (*n* = 5 for each group). Group means were compared by Student’s *t*‐test. Data shown are means ± SEM. *P < 0.05. ns, no statistical difference.

To further explore the regulation of CYP1A1 in ALI, we assessed major pro‐inflammatory factors in lungs of LPS‐induced CYP1A1+/+ and CYP1A1−/− mice. Following LPS treatment, the levels of TNF‐α, IL‐1β, IL‐6, and NO were elevated in lung tissue homogenates of LPS‐induced CYP1A1+/+ mice compared to PBS‐treated mice, while these upregulations were significantly amplified by CYP1A1 deficiency (Fig. 3A). To determine the source of NO, lung tissue was harvested for iNOS assessment by western blot analysis and immunohistochemistry. As shown in Fig. 3B,C, in response to LPS treatment the protein level of iNOS was increased in CYP1A1+/+ mice and potentiated by CYP1A1 deficiency. LPS‐induced ALI is typically accompanied by an increase in alveolar capillary membrane permeability, an enrichment of airspace exudate protein, and neutrophil infiltration. The protein concentration of BALF was increased in CYP1A1 deficient mice (Fig. 3D). MPO activity, a classic marker of neutrophil infiltration, was elevated in lungs of LPS‐induced CYP1A1−/− mice compared to LPS‐treated CYP1A1+/+ mice (Fig. 3D). Consistent with these results, levels of TNF‐α, IL‐1β, IL‐6, and NO were also increased in BALF from CYP1A1−/− mice (Fig. 3E). These data suggest CYP1A1 to be an important surrogate for pro‐inflammatory factor regulation in LPS‐induced ALI.

### CYP1A1 deficiency promotes NF‐κB activation in LPS‐induced ALI

**Fig. 4:**
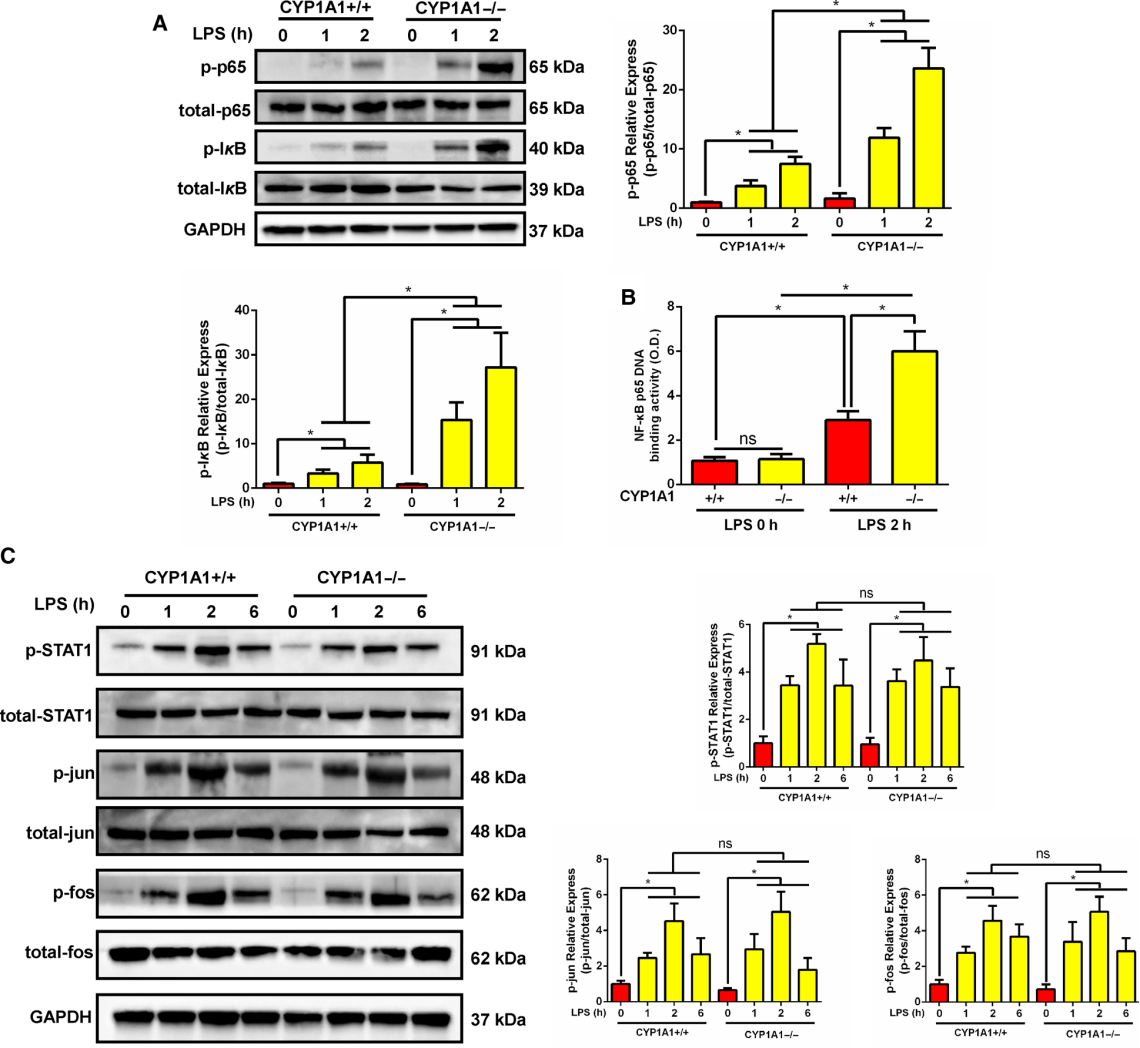
CYP1A1 deficiency promoted NF‐κB activation in LPS‐induced ALI. (A‐C) CYP1A1+/+ and CYP1A1−/− mice were intraperitoneally injected with PBS or LPS (15 mg·kg^−1^) for 1, 2, or 6 h. (A) The phosphorylation and total levels of p65 and IκB‐α in lung homogenates were analyzed by western blot after 1 and 2 h of LPS treatment and quantified by densitometry. (B) The DNA‐binding activity of p65 was determined with a TransAM p65 transcription factor ELISA kit (*n* = 5 for each group). (C) The phosphorylation and total levels of STAT1 and AP‐1 (jun and fos) in lung homogenates were examined at the indicated times and quantified by densitometry. Group means were compared by Student's *t*‐test. Data shown are means ± SEM. *P < 0.05. ns, no statistical difference.

As a critical regulator of inflammation, the NF‐κB pathway plays an important role in ALI. To assess the effect of CYP1A1 on NF‐κB activation, we investigated the phosphorylation and DNA‐binding activity of NF‐κB p65 in LPS‐induced ALI. By western blot analysis, CYP1A1 deficiency was found to promote the phosphorylation of NF‐κB p65 in lung tissue after 1 and 2 h of LPS treatment, with concomitant enhanced degradation of IκB‐α (Fig. 4A). Consistent with western blot results, CYP1A1 knockout mice had significantly increased NF‐κB p65 DNA‐binding activity compared to CYP1A1+/+ mice (Fig. 4B). Activator protein 1 (AP‐1, jun, and fos) and signal transducer and activator of transcription 1 (STAT1) are two critical regulators of pro‐inflammatory cytokines and iNOS expression. As shown in Fig. 4C, CYP1A1 knockout had no significant effect on the activation of AP‐1 or STAT1. Taken together, these results suggest that for CYP1A1 deficiency, induced pro‐inflammatory responses result from activation of NF‐κB p65 but not AP‐1 or STAT1.

### CYP1A1 deficiency‐induced pro‐inflammatory responses are NF‐κB‐dependent in ALI

**Fig. 5:**
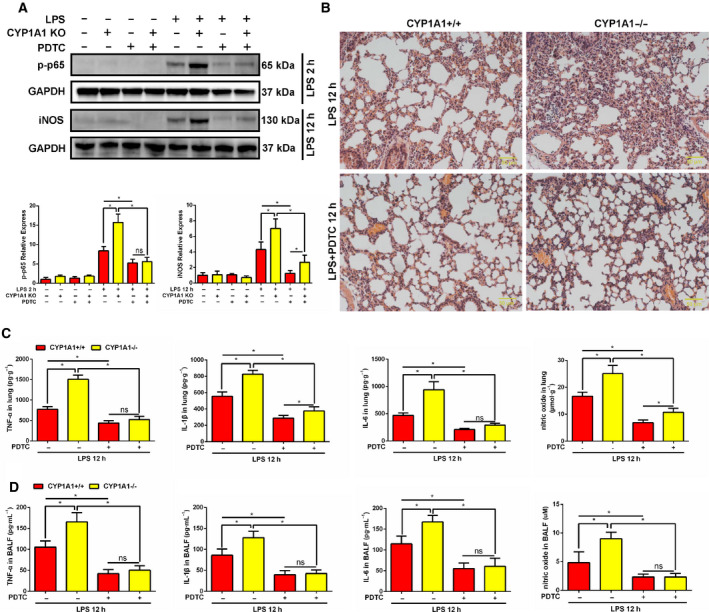
CYP1A1 deficiency‐induced pro‐inflammatory responses in ALI are NF‐κB‐dependent. (A–D) CYP1A1+/+ and CYP1A1−/− mice were pretreated with a NF‐κB inhibitor, PDTC (100 mg·kg^−1^, intraperitoneal injection), for 2 h and then intraperitoneally injected with PBS or LPS (15 mg·kg^−1^) for the indicated time. (A) Phosphorylation level of p65 (LPS 2 h) and total iNOS (LPS 12 h) was detected by western blot and quantified by densitometry. (B) Lung tissue H&E stained. Bar: 50 μm. (C) TNF‐α, IL‐1β, IL‐6, and NO levels in lung homogenates (*n* = 5 for each group). (D) TNF‐α, IL‐1β, IL‐6, and NO levels in BALF (*n* = 5 for each group). Group means were compared by Student's *t*‐test. Data are shown as means ± SEM. *P < 0.05. ns, no statistical difference.

Given that CYP1A1 knockout promotes NF‐κB activation, we used an NF‐κB selective inhibitor, PDTC, to determine whether regulation of CYP1A1 is NF‐κB‐dependent in ALI. As shown in Fig. 5A, PDTC treatment significantly abolished NF‐κB activation and iNOS expression associated with CYP1A1 knockout. Using H&E staining, we confirmed PDTC to partially relieve damage caused by CYP1A1 deficiency as evidenced by reduced neutrophil infiltration and improved lung architecture (Fig. 5B). Furthermore, the excessive levels of TNF‐α, IL‐1β, IL‐6, and NO in lung (Fig. 5C) and BALF (Fig. 5D), induced by CYP1A1 deficiency, were blocked by PDTC treatment. These results confirm that CYP1A1 knockout‐induced pro‐inflammatory cytokine and iNOS production are NF‐κB‐dependent.

### Systemic CYP1A1 knockout in mice intensifies organ damage in response to LPS

**Fig. 6:**
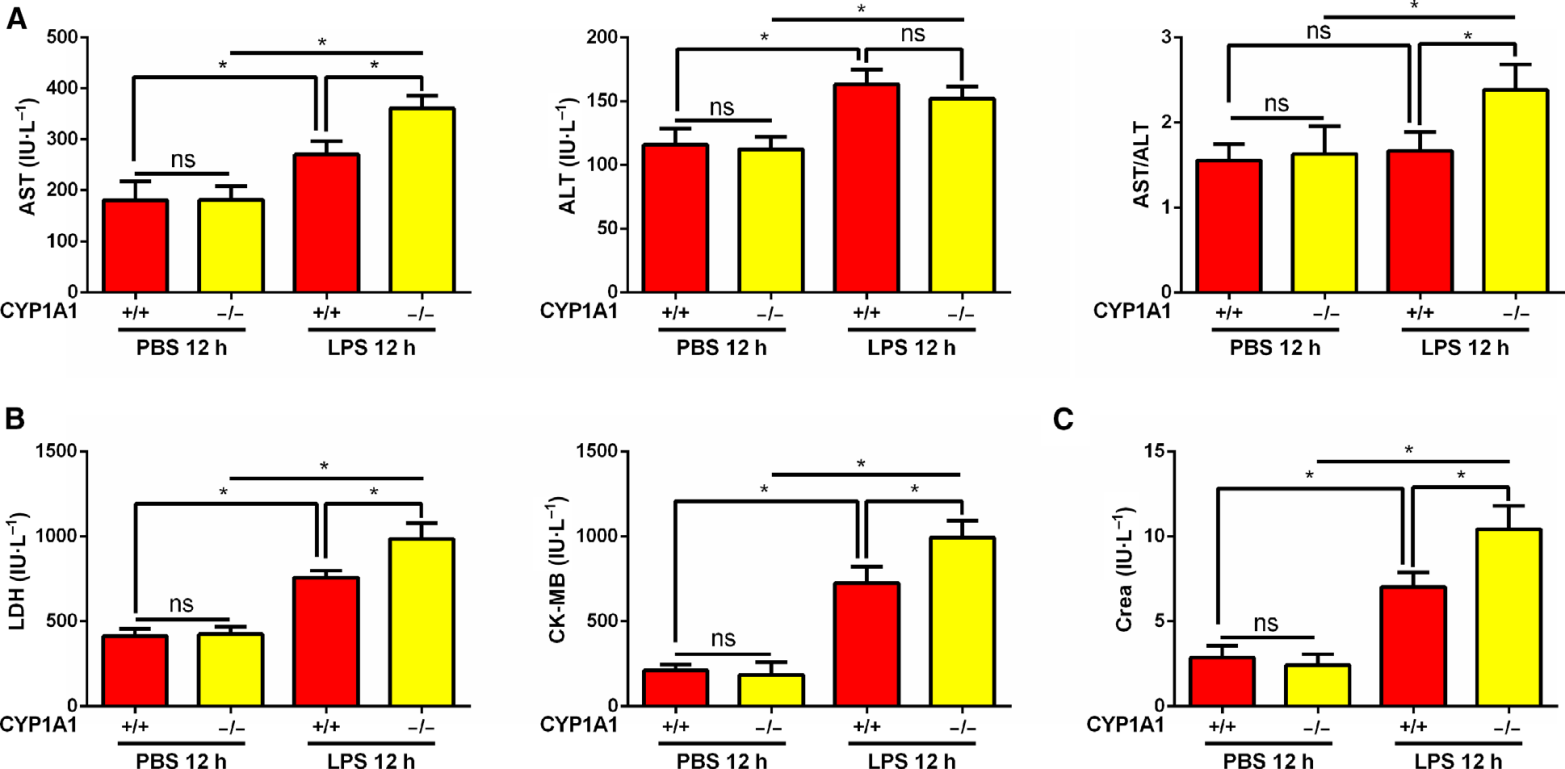
Systemic CYP1A1 knockout intensifies murine organ damage in response to LPS. (A‐C) CYP1A1+/+ and CYP1A1−/− mice were intraperitoneally injected with PBS or LPS (15 mg·kg^−1^) for 12 h. Serum was assessed for hepatic (A), cardiac (B), and renal (C) functional markers (*n* = 5 for each group). Group means were compared by Student’s *t*‐test. Data are shown as means ± SEM. *P < 0.05. ns, no statistical difference.

To further investigate the role of CYP1A1 in the progression of systemic inflammation, serum was collected from LPS‐induced CYP1A1+/+ and CYP1A1−/− mice and evaluated for markers of hepatic, cardiac, and renal function. We found AST levels and the AST/ALT ratio to be moderately elevated in LPS‐induced CYP1A1−/− compared to CYP1A1+/+ mice. There was no significant change in ALT levels (Fig. 6A). LDH and CK‐MB, two important biomarkers of cardiac function, were also increased in LPS‐induced and CYP1A1‐deficient mice (Fig. 6B). For renal function, levels of Crea were increased in LPS‐induced CYP1A1−/− compared to CYP1A1+/+ mice (Fig. 6C). These data imply a possible role for CYP1A1 in LPS‐induced systemic responses.

### CYP1A1 deficiency intensifies *E*.* coli*‐induced lung injury in septic mice

**Fig. 7:**
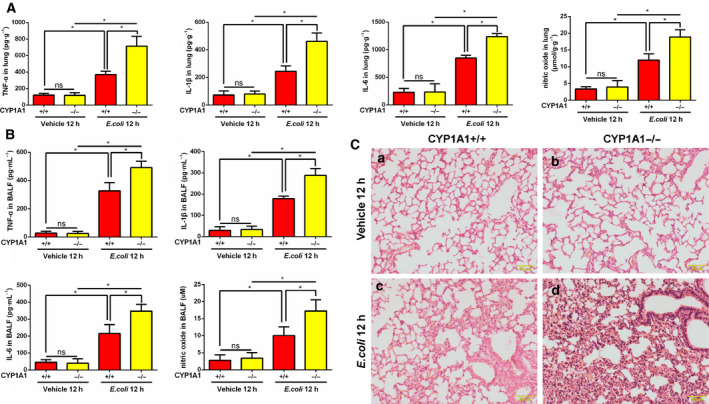
CYP1A1 deficiency intensifies *E*.* coli*‐induced lung injury in septic mice. (A‐C) CYP1A1+/+ and CYP1A1−/− mice were intratracheally instilled with *E*.* coli* (CFU 5 × 10^8^/40 μL) or vehicle for 12 h. (A) TNF‐α, IL‐1β, IL‐6, and NO levels were measured in lung homogenates of treated mice by ELISA (*n* = 5 for each group). (B) TNF‐α, IL‐1β, IL‐6, and NO levels assessed in BALF (*n* = 5 for each group). (C) Sections of lungs were stained with H&E. Bar: 50 μm. Group means were compared by Student’s *t*‐test. Data shown are means ± SEM. *P < 0.05. ns, no statistical difference.

To further investigate the function of CYP1A1 in actual respiratory infection, CYP1A1+/+ and CYP1A1−/− mice were intratracheally instilled with *E*.* coli* or vehicle. As shown in Fig. 7A, the levels of TNF‐α, IL‐1β, IL‐6, and NO in lung tissues were elevated by *E*.* coli* treatment, while CYP1A1 knockout increased the expression of these inflammatory cytokines compared to CYP1A1+/+ group. In accordance with these results, levels of TNF‐α, IL‐1β, IL‐6, and NO were also increased in BALF from *E*.* coli*‐induced CYP1A1−/− mice (Fig. 7B). Using H&E staining, morphologic alterations were observed in *E*.* coli*‐induced lung injury group, including extensive alveolar wall thickening, neutrophil infiltration, and alveolar collapse, while CYP1A1 deficiency amplified these histological alterations (Fig. 7C). These results preliminarily confirmed the regulation effect of CYP1A1 in respiratory infection.

## Discussion

In this study, CYP1A1 was found to be highly expressed in the lungs, liver, and kidney of mice treated with LPS. Interestingly, we found that CYP1A1 increase in LPS‐induced lungs was AhR‐independent. Emerging studies have reported upregulation of lung CYP1A1 in response to multiple stimuli. A previous study showed lung CYP1A1 to be increased in lungs following hyperoxic challenge [10]. Further, CYP1A1 protein levels were elevated in the lungs of mice in response to incense smoke [11]. Diesel exhaust particles, a common air pollutant, promote CYP1A1 mRNA levels in human bronchial epithelial cells, accompanied by pro‐inflammatory factor expression [16]. The results of this study are consistent with these previous findings. Although CYP1A1 expression is mainly regulated by AhR [5], we found that CYP1A1 was upregulated in the lungs of LPS‐induced AhR−/− mice. We also observed that CYP1A1 deficiency had no effect on AhR expression in the lung. These results suggest CYP1A1 to be an inflammatory regulator in an AhR‐independent manner. As a specific ligand of TLR4, LPS promoted CYP1A1 expression in the lung and we found that TLR4 knockout significantly reduced LPS‐induced CYP1A1 expression. It has been reported that LPS can activate AhR via a TLR4‐NF‐κB pathway [17]. Herein, we found CYP1A1 expression in LPS‐induced lungs to be AhR‐independent, suggesting that other pathways may be involved in this regulatory process. For instance, glycine methyl transferase has been shown to upregulate CYP1A1 [18], while Nrf2, Oct‐1, and C/EBP have been shown to suppress CYP1A1 [19, 20]. However, those results were not examined in LPS‐induced lung tissue. Taken together, these results provide a potential strategy for the regulation of CYP1A1 expression in ALI.

Excessive pro‐inflammatory mediator production is a significant cause of ALI tissue damage [21, 22, 23, 24]. Thus, the aim of this study was to identify a novel target for treatment of ALI, a target that would reduce a pro‐inflammatory response. Emerging evidence has demonstrated CYP1A1 to be involved in several inflammatory reactions through differential regulation. Activation of CYP1A1 decreased pro‐inflammatory mediator production by *Mycoplasma‐*induced pulmonary alveolar macrophages through targeting of PPAR‐γ [9]. CYP1A1 knockout augmented lung injury caused by hyperoxia, but the underlying mechanism was not identified [10]. CYP1A1 overexpression in bovine mammary epithelial cells ameliorated LPS‐induced inflammatory responses by suppressing NF‐κB activation [25]. However, there are several studies that report CYP1A1 to promote inflammatory responses. LTB_4_, an inflammatory factor, was shown to be decreased in neutrophils isolated from zymosan‐induced CYP1A1 knockout mice [26]. It has been also demonstrated that systemic CYP1A1 overexpression in mice significantly increased mouse mortality following *Citrobacter rodentium* infection [27]. These results suggest that regulation of CYP1A1 during inflammation is species‐, tissue‐, and cell type‐specific. We found that MPO activity, a marker of neutrophil infiltration, was increased in lungs of LPS‐induced CYP1A1−/− mice, which is similar to a previous study that reported a similar phenomenon in the lungs of CYP1A1−/− mice in response to hyperoxia [10], but the mechanistic basis for this phenomenon is unknown. Since LPS is a key surrogate to trigger amounts of cascade reactions in ALI, such as cytokine storm, lung edema, and systematic shock, we aimed to explore whether CYP1A1 was involved in these LPS‐induced alterations. In this study, we found that CYP1A1 deficiency increased TNF‐α, IL‐1β, IL‐6, and NO levels in LPS‐triggered ALI, which is consistent with several lines of previous evidence. Our data also demonstrated that CYP1A1 deficiency augmented LPS‐induced ALI by increasing lung edema and neutrophil infiltration, as well as destruction of lung architecture. Furthermore, we showed for the first time that systemic CYP1A1 knockout intensified liver, heart, and kidney damage in response to LPS challenge. Moreover, we also confirmed that CYP1A1 aggravated *E*.* coli*‐induced lung injury by increasing the expression of inflammatory cytokines and amplifying lung histological alterations. These data suggest CYP1A1 to be a novel target for ALI treatment.

NF‐κB is a key regulator of inflammatory factor production and is involved in the progression of ALI. In the current study, we found activation of NF‐κB to be increased by CYP1A1 knockout in LPS‐induced ALI. PDTC treatment reversed the pro‐inflammatory response caused by CYP1A1 knockout, revealing a novel axis, CYP1A1‐NF‐κB, in ALI. Epoxyeicosatrienoic acids (EETs) are a series of products produced by CYP1A1 epoxidase activity [28]. It has been reported that EETs suppress pro‐inflammatory mediators in ALI by targeting NF‐κB [29, 30, 31, 32], indicating that the pro‐inflammatory response caused by CYP1A1 deficiency in ALI may be EET‐related. However, it is worth noting that 12S‐hydroxy‐5Z, 8Z, 10E, 14Z‐eicosatetraenoic acid, which is produced by CYP1A1 hydroxylase activity [28], is an inducer of NF‐κB [33]. Thus, further investigation is necessary to determine the underlying mechanistic basis for CYP1A1 and NF‐κB activation. NO expression in LPS‐induced ALI is mainly produced by iNOS activity, which is controlled by NF‐κB activation [34]. By use of PDTC, we confirmed that overexpression of iNOS, in LPS‐induced ALI with concomitant CYP1A1 deficiency, is NF‐κB‐dependent. It has been reported that iNOS expression was elevated by CYP1A1 inducer in rat polymorphonuclear leukocytes with a [Ca^2+]^i‐dependent manner, while CYP1A1 inhibitor abolished this effect [35]. However, this regulation mediated by CYP1A1 inducer or inhibitor may be related to a nonspecific effect. Another study reported that aminoguanidine, an iNOS inhibitor, suppressed CYP1A1 enzyme activity in diesel exhaust particle‐induced lung inflammation, while the direct effect of CYP1A1 in regulating iNOS expression was not identified [36]. Inconsistent with the previous study [35], we showed for the first time that CYP1A1 deficiency directly increased iNOS expression in LPS‐induced lung injury, suggesting that regulation of CYP1A1 on iNOS expression is cell type‐specific.

Taken together, these preliminary data reveal an underlying mechanistic role for CYP1A1 in regulation of pro‐inflammatory responses in LPS‐induced ALI.

There are several limitations to this study. Although we observed that LPS‐induced lung CYP1A1 overexpression was AhR‐independent, it is unknown which transcription factors mediate CYP1A1 expression in the lungs of AhR−/− mice. Although we showed a relationship between CYP1A1 and NF‐κB activation in LPS‐induced lungs, the identification of a specific mechanism and the cell type involved require further investigation. We demonstrated an *in vivo* pro‐inflammatory effect of CYP1A1 deficiency in LPS‐induced mice as evidenced by liver, heart, and kidney functional damage. However, the effect of CYP1A1 on pathology and mortality in mice challenged with polymicrobial sepsis, trauma, or LPS requires further investigation. These issues will be addressed in our future studies. Nevertheless, this study demonstrates for the first time that a CYP1A1‐NF‐κB pathway augmented LPS‐induced ALI and revealed a novel role for CYP1A1 in the systemic inflammatory response.

## Conclusions

In conclusion, we observed an increase of CYP1A1 in LPS‐induced ALI in an AhR‐independent manner. Further, TLR4 knockout abolished CYP1A1 overexpression. CYP1A1 deficiency elevated TNF‐α, IL‐1β, IL‐6, and NO levels in lung tissues of septic mice by overactivation of NF‐κB and iNOS. Furthermore, we confirmed that deletion of CYP1A1 increased lung edema, neutrophil infiltration, and alveolar structural destruction as evidenced by H&E staining in response to LPS or *E*.* coli* challenge. In a preliminary manner, we demonstrated that systemic CYP1A1 knockout induced multiple organ damage in response to LPS. These results suggest that CYP1A1 may be a potential therapeutic target for treatment of LPS‐triggered ALI.

## Conflict of interest

The authors have no conflict of interest to declare.

## Author contributions

L‐XT and XT performed most of the experiments, analyzed the research data, and wrote the manuscript. JW and WZ helped with animal experiments. KL and WM helped with cellular experiments. TC and H‐PL contributed to experimental design. H‐PL and J‐YZ designed experiments, interpreted data, and supervised the work.

## Data Availability

The datasets used and/or analyzed during the current study are available from the corresponding author on reasonable request.
